# Association Between Local Ascending Aortic Elastic Properties and Left Ventricular Global Longitudinal Strain in Patients with Multiple Sclerosis: A Cross-Sectional Case–Control Echocardiographic Study

**DOI:** 10.3390/diagnostics16203385

**Published:** 2026-10-18

**Authors:** Elçin Özdemir Tutar, Pınar Öztürk

**Affiliations:** 1 Department of Cardiology, Ankara Yıldırım Beyazıt University Yenimahalle Training and Research Hospital, Ankara 06370, Türkiye; 2 Department of Neurology, Ankara Yıldırım Beyazıt University Yenimahalle Training and Research Hospital, Ankara 06370, Türkiye; pnr_akyz@hotmail.com

**Keywords:** multiple sclerosis, aortic stiffness, aortic distensibility, aortic strain, global longitudinal strain, speckle-tracking echocardiography, ventricular–arterial interaction, subclinical cardiovascular disease

## Abstract

**Background/Objectives:** Multiple sclerosis (MS) has been associated with subclinical cardiovascular involvement, including impaired myocardial deformation and increased arterial stiffness. However, the local elastic properties of the ascending aorta and their relationship with subclinical left ventricular (LV) longitudinal dysfunction remain insufficiently characterized in MS. **Methods:** In this prospective cross-sectional study, 67 adults with MS and 67 age-matched controls without cardiovascular disease underwent standardized transthoracic echocardiography. Aortic strain, distensibility and stiffness index (β) were derived from M-mode ascending aortic diameters and simultaneous brachial blood pressure, and LV global longitudinal strain (GLS) was measured by two-dimensional speckle-tracking echocardiography. Multivariable linear regression was utilized to assess associations. **Results:** LV ejection fraction was similar between groups, while LV GLS was less negative in MS (−17.49 ± 1.81% vs. −19.85 ± 1.80%, *p* < 0.001). Patients with MS had lower aortic strain (7.54 ± 3.66% vs. 12.91 ± 3.84%) and distensibility (3.81 [2.23–6.06] vs. 5.95 [4.90–9.26] × 10^−3^ mmHg^−1^) and a higher stiffness index (4.77 [3.10–7.10] vs. 3.44 [2.31–4.05]) (all *p* < 0.001). After adjustment for age, sex, and body mass index, MS remained independently associated with aortic strain, distensibility, and stiffness index. Within the MS group, aortic strain was independently associated with LV GLS after adjustment for age, sex, body mass index, Expanded Disability Status Scale score and LDL cholesterol (B = −0.276, standardized β = −0.558, *p* < 0.001). LV GLS was independently associated with log-transformed distensibility (B = −1.021, *p* = 0.011) and stiffness index (B = 1.095, *p* = 0.014). **Conclusions:** Patients with MS show concurrent, inter-related impairment of local ascending aortic elasticity and LV longitudinal deformation despite preserved ejection fraction. Further studies are needed to establish the temporal sequence and prognostic relevance of these findings.

## 1. Introduction

Multiple sclerosis (MS) is a chronic immune-mediated disease of the central nervous system characterized by focal inflammatory demyelination and progressive neurodegeneration [1,2]. Approximately 2.8 million people are affected worldwide, corresponding to an estimated global prevalence of 35.9 per 100,000 population [3]. Morbidity and management of MS have become increasingly important as available treatment modalities have contributed to longer overall survival. Cardiovascular comorbidity is clinically relevant in this population; hypertension and hyperlipidemia are among the most common vascular comorbidities and increase with age [4], while large population-based cohorts have documented an elevated risk of myocardial infarction, heart failure, and stroke after an MS diagnosis that is not fully explained by conventional risk factors [5,6]. More recently, vascular disease was reported to account for 16.7% of deaths among deceased individuals with MS, further emphasizing the cardiovascular burden of the disease [7]. Vascular comorbidity is, in turn, associated with neurological disability, suggesting a bidirectional relationship between vascular health and the disease course [8,9].

Arterial stiffness is an established, independent predictor of cardiovascular events and mortality in the general population [10,11,12]. The proximal aorta buffers the outcome of left ventricular (LV) ejection, and loss of this cushioning function increases the pulsatile load imposed on the ventricle and the microcirculation [13,14]. In MS, a recent meta-analysis found overall impairment of vascular function compared with healthy controls, with differences observed for indices of arterial stiffness [15]. Individual studies using applanation tonometry, pulse contour analysis or oscillometric devices have reported higher pulse wave velocity, reduced arterial compliance and greater wave reflection in MS [16,17,18,19,20]. Proposed factors contributing include physical inactivity, autonomic dysfunction, endothelial dysfunction, oxidative stress and systemic inflammation, and routinely accepted cardiometabolic risk factors [17,18,21,22,23,24,25,26,27,28,29].

Cardiac involvement in MS may remain clinically silent. LV ejection fraction (LVEF) is an insensitive measure of early myocardial dysfunction, while global longitudinal strain (GLS) derived from two-dimensional speckle-tracking echocardiography (2D-STE) may detect subclinical longitudinal dysfunction [30,31,32]. Early echocardiographic and tissue Doppler studies suggested subtle LV dysfunction in MS, and more recent imaging studies have reported less negative LV GLS in patients with MS, with an inverse relationship between GLS and the Expanded Disability Status Scale (EDSS) score [33,34,35,36]. As GLS is influenced by age, sex, loading conditions and specific software, it should be interpreted as a marker of subclinical longitudinal dysfunction rather than as a disease-specific indicator of myocardial injury [37,38].

Myocardial and vascular abnormalities are physiologically coupled. In a large community-based cohort, greater aortic stiffness was independently associated with worse LV GLS [39]. Consensus documents emphasize that sensitive myocardial (e.g., GLS) and arterial (e.g., stiffness) markers may reveal abnormal ventricular–arterial interaction before overt disease develops [40]. In MS, tonometry-based studies have related central pulse pressure to walking impairment and have described higher arterial and ventricular elastance with preserved coupling [41,42]. Mincu et al. evaluated myocardial deformation together with arterial stiffness and endothelial function in a single MS cohort and observed impaired biventricular deformation, yet no significant differences in the vascular parameters were observed [35].

Aortic strain, aortic distensibility and the aortic stiffness index (β) are simple, noninvasive indices of the local elastic behavior of the proximal ascending aorta that can be derived from M-mode measurements of systolic and diastolic aortic diameters combined with brachial blood pressure [43,44,45,46,47,48]. Echocardiographic aortic distensibility correlates closely with invasive measurements, and these indices deteriorate progressively with age in normotensive adults, with reductions in ascending aortic strain and distensibility being among the earliest manifestations of vascular aging [44,49,50]. Because they describe local ascending aortic behavior, they complement other measures, including carotid–femoral pulse wave velocity or augmentation index [13]. To our knowledge, these local echocardiographic indices have not been evaluated in MS.

Accordingly, the aims of this study were to compare echocardiographically derived local ascending aortic elastic properties and LV GLS between patients with MS and age-matched controls without cardiovascular disease, to examine the possible associations between the aortic elastic indices and LV GLS within the MS group, and to explore the relationships of these indices with MS-related clinical characteristics, including EDSS score and disease duration.

## 2. Materials and Methods

This prospective, observational, cross-sectional case–control study was conducted between June and July 2026 at a tertiary center. The study protocol was approved by the local ethics committee, titled “Ankara Yıldırım Beyazıt University Yenimahalle Training and Research Hospital Non-Interventional Clinical Research Ethics Committee,” under approval no. E-2026-59 and dated 18 June 2026. The study was conducted in accordance with the principles of the Declaration of Helsinki, and all participants provided written informed consent before enrolment.

Patients aged ≥18 years with an established diagnosis of MS according to the 2024 revised McDonald diagnostic criteria who presented to the Neurology Outpatient Clinic for routine follow-up during the study period were invited to undergo a standardized cardiovascular evaluation, irrespective of the presence of cardiac symptoms. The control group comprised age-matched individuals who presented to the Cardiology Outpatient Clinic during the same period for routine evaluation, had no active cardiovascular symptoms and no significant abnormalities on physical examination, blood pressure assessment, 12-lead electrocardiography or transthoracic echocardiography. Matching was performed at the group level for age; no matching was performed for sex.

To restrict the analysis to participants without overt LV systolic dysfunction, individuals with an LVEF < 50% were excluded. Further cardiovascular exclusion criteria were known coronary artery disease, previous myocardial infarction or coronary revascularization, moderate or severe valvular heart disease, congenital heart disease, cardiomyopathy, atrial fibrillation or atrial flutter, and other clinically significant arrhythmias precluding reliable echocardiographic or strain analysis. Additional exclusion criteria applicable to both groups were severely uncontrolled blood pressure at the study visit (systolic ≥ 180 mmHg and/or diastolic ≥ 110 mmHg), active infection, active malignancy, connective tissue disease, vasculitis, bicuspid aortic valve, aortic aneurysm or other significant aortic pathology, and inadequate echocardiographic image quality preventing reliable measurement of LV GLS or ascending aortic diameters. In the MS group, additional laboratory-based exclusion criteria were uncontrolled diabetes mellitus (HbA1c ≥ 7.5%) and chronic kidney disease (eGFR < 60 mL/min/1.73 m^2^). In controls, uncontrolled diabetes mellitus was excluded based on clinical assessment, as HbA1c values were not systematically recorded. Baseline serum creatinine results were available in the medical records and were within the laboratory reference range, but were not included in the study dataset. HbA1c- and eGFR-based exclusion thresholds were not systematically applied to controls. Hyperlipidemia was defined as a known diagnosis of hyperlipidemia or current statin use, based on medical history and medication review. No LDL cholesterol threshold was used for classification.

Demographic characteristics, cardiovascular risk factors, medical history, and current medications were recorded for all participants.

Body mass index (BMI) was calculated as weight in kilograms divided by the square of height in meters. In the MS group, disease phenotype, disease duration, EDSS score, relapse history, time since the last relapse, previous corticosteroid treatment for relapse, and current disease-modifying therapy were also documented. Disease duration was defined as the interval between the date of MS diagnosis and the echocardiographic examination, and time since the last relapse as the interval between the most recent documented relapse and the examination. The neurologist responsible for the patients’ clinical follow-up assigned EDSS scores. In the MS group, fasting venous blood samples were obtained for routine laboratory assessment. Laboratory analyses included lipid profile, glucose, glycated hemoglobin, creatinine, hemoglobin, C-reactive protein, and thyroid-stimulating hormone.

Brachial systolic (SBP) and diastolic blood pressure (DBP) were measured in the right arm with a validated automated oscillometric device (OMRON M2, HEM-7121-E; OMRON Healthcare Co., Ltd., Kyoto, Japan), while the participant rested in the supine position simultaneously with the echocardiographic acquisition of ascending aortic diameters. Three consecutive readings were obtained under identical resting conditions, and the mean of the second and third readings was used for the calculation of the aortic elastic indices. Pulse pressure was calculated as SBP − DBP.

Comprehensive transthoracic echocardiography was performed with a Philips Affiniti 50 ultrasound system (Philips Healthcare, Andover, MA, USA) with continuous electrocardiographic monitoring, with the participant in the left lateral decubitus position. Two-dimensional, M-mode, pulsed-wave Doppler and tissue Doppler measurements were obtained from standard parasternal and apical views according to the recommendations of the American Society of Echocardiography and the European Association of Cardiovascular Imaging [51]. LV dimensions and wall thicknesses, left atrial volume index (LAVI), LV mass index (LVMI), transmitral inflow velocities, mitral annular tissue Doppler velocities, right ventricular (RV) dimensions and function, tricuspid annular plane systolic excursion (TAPSE), RV tissue Doppler systolic velocity (S′), and valvular morphology and function were assessed. LV end-diastolic and end-systolic volumes were measured from the apical four- and two-chamber views, and LVEF was calculated using the modified biplane Simpson method [51]. When an adequate tricuspid regurgitation signal was available, systolic pulmonary artery pressure (sPAP) was estimated from the peak tricuspid regurgitation velocity using the simplified Bernoulli equation with the addition of estimated right atrial pressure. Pericardial effusion was defined echocardiographically as an echo-free space between the visceral and parietal pericardial layers. In all affected participants, the effusion was small, sheet-like, did not exceed 10 mm in maximal diastolic separation, and was not associated with echocardiographic signs of cardiac tamponade.

LV myocardial deformation was assessed by 2D-STE in accordance with current consensus recommendations [30]. Apical four-chamber, two-chamber and long-axis views were acquired at a frame rate of 50–70 frames/s. Offline LV strain analysis was performed using the AutoStrain LV application on the vendor-independent TOMTEC-ARENA platform (version 2.40; TomTec Imaging Systems GmbH, Unterschleissheim, Germany) for all participants. After identification of the endocardial border, the software tracked myocardial speckles automatically throughout the cardiac cycle; tracking quality was inspected visually in every segment, and the region of interest was adjusted manually when required. Segmental longitudinal strain values were evaluated on a 17-segment model, and LV GLS was computed from the three apical views. Strain is reported using the signed convention, with myocardial shortening represented by negative values.

The local elastic properties of the ascending aorta were assessed by two-dimensionally guided M-mode echocardiography from the parasternal long-axis view, following previously described methodology [44,48,52]. The M-mode cursor was positioned perpendicular to the walls of the proximal tubular ascending aorta approximately 3 cm above the aortic valve. Systolic (AoS) and diastolic (AoD) aortic diameters were measured with an inner-edge-to-inner-edge convention. For each participant, a single cardiac cycle with adequate visualization of the anterior and posterior aortic walls and free of ectopic beats was selected. AoS was defined as the maximal aortic diameter during systolic expansion and AoD was measured at end-diastole at the peak of the QRS complex on the simultaneously recorded electrocardiogram.

Aortic strain, aortic distensibility and the aortic stiffness index (β) were calculated from AoS, AoD and the simultaneously recorded brachial SBP and DBP using established formulas [43,44,45,46,49]:

Aortic strain (%) = 100 × (AoS − AoD)/AoD(1)

Aortic distensibility (mmHg^−1^) = 2 × [(AoS − AoD)/AoD]/(SBP − DBP) (2)

Aortic stiffness index (β) = ln(SBP/DBP)/[(AoS − AoD)/AoD](3)

The primary outcomes were the between-group differences in the three local ascending aortic elastic indices (aortic strain, aortic distensibility and aortic stiffness index). Secondary outcomes were the between-group difference in LV GLS, the associations between LV GLS and the aortic elastic indices, and, within the MS group, the associations of LV GLS and the aortic elastic indices with MS-related clinical characteristics and laboratory parameters.

Reproducibility of aortic measurements was assessed in 30 randomly selected participants. The first observer repeated the measurements two weeks after the initial assessment to evaluate intraobserver reproducibility. A second observer independently performed measurements on the same stored echocardiographic images to assess interobserver reproducibility. During reassessment, both observers were blinded to MS/control status and previous measurement results. Aortic strain, distensibility, and stiffness index β were recalculated separately from each observer’s independently measured systolic and diastolic aortic diameters, using the same recorded blood pressure values for each participant. Agreement was assessed using single-measure, two-way absolute-agreement intraclass correlation coefficients [ICC(A,1)] and Bland–Altman analysis. The 95% confidence intervals for ICCs were obtained using 10,000 participant-level bootstrap resamples.

### Statistical Analysis

Statistical analyses were conducted using IBM SPSS Statistics version 29.0 (IBM Corp., Armonk, NY, USA). The distribution of continuous variables was assessed using histograms, Q–Q plots, and the Shapiro–Wilk test. Normally distributed variables were presented as mean ± standard deviation (SD), and non-normally distributed variables as median (interquartile range, IQR). Student’s t-test or the Mann–Whitney U test was used for continuous variables, and Pearson’s chi-square test or Fisher’s exact test was used for categorical variables. Between-group effect sizes for the principal normally distributed outcomes were expressed as Cohen’s d.

Separate multivariable linear regression models were prepared for relevant outcomes. Multicollinearity was assessed with tolerance and variance inflation factors, and the linearity, normality and homoscedasticity of residuals and the presence of influential observations were evaluated with residual and diagnostic plots. All tests were two-sided, and *p* < 0.05 was considered statistically significant.

Age, sex, and BMI were selected a priori as clinically relevant covariates potentially affecting myocardial deformation and arterial elastic properties. Hyperlipidemia and systolic blood pressure were subsequently evaluated in sensitivity analyses because of their potential cardiovascular confounding effects and the observed between-group imbalance in hyperlipidemia.

To address multiple testing, a Bonferroni-adjusted significance threshold of *p* < 0.0125 was applied to the four principal between-group echocardiographic outcomes (LV GLS, aortic strain, aortic distensibility, and aortic stiffness index). For the 20 exploratory within-MS correlations of clinical and laboratory variables with LV GLS and the aortic elastic indices, *p* values were additionally adjusted using the Benjamini–Hochberg false discovery rate procedure. FDR-adjusted *p* values are presented in Supplementary Table S2.

Sample Size Calculation: The sample size calculation was based on the expected between-group difference in LV global longitudinal strain (GLS). In a previous study by Demir et al., patients with MS had significantly impaired GLS compared with controls (−17.05 ± 1.33% vs. −18.99 ± 1.08%, respectively), corresponding to a large standardized effect size (Cohen’s d ≈ 1.58) [36]. To provide a more conservative estimate and avoid overestimating the expected effect, an effect size of d = 0.60 was prespecified for the present study. Using G*Power version 3.1, with a two-sided α of 0.05, 80% power, and a 1:1 allocation ratio, at least 45 participants per group were required. Allowing for potential exclusions and incomplete or inadequate echocardiographic data, recruitment of at least 50 participants per group was planned. A total of 75 patients with MS were initially assessed for eligibility. Eight patients were excluded: three because of a left ventricular ejection fraction <50%, three because of uncontrolled hypertension, and two because of connective tissue disease. Consequently, 67 patients with MS were included in the final analysis.

## 3. Results

The final study population comprised 134 participants (67 patients with MS and 67 controls). Mean age was similar in the two groups (41.51 ± 10.80 vs. 42.36 ± 9.67 years, *p* = 0.632). Women were more prevalent in the MS group (86.6% vs. 65.7%, *p* = 0.005), and BMI was lower in patients with MS (24.1 [22.3–26.6] vs. 24.9 [23.3–28.7] kg/m^2^, *p* = 0.020). Systolic and diastolic blood pressure and the prevalence of hypertension and diabetes mellitus did not differ significantly between the groups, whereas hyperlipidemia was more frequent in patients with MS (23.9% vs. 7.5%, *p* = 0.009). The use of antihypertensive medications and statins did not differ significantly between the groups (14.9% vs. 16.4% and 6.0% vs. 7.5%, respectively; both *p* = 1.000). In the MS group, the median disease duration was 12.0 (5.0–20.0) years, the median time since the last relapse was 3.0 (1.0–9.0) years, and the median EDSS score was 1.0 (1.0–2.0). Sixty-one patients (91.0%) had previously received corticosteroid treatment for a relapse (Table 1).

LVEF was preserved and did not differ significantly between the two groups (62.0 [59.0–67.0]% in controls vs. 65.0 [59.0–67.0]% in patients with MS, *p* = 0.872), and there were no significant between-group differences in LAVI, LVMI, TAPSE, RV S′, or estimated sPAP. LV end-diastolic and end-systolic diameters were smaller in the MS group (both *p* < 0.001). Pericardial effusion was more frequent in patients with MS than in controls (20.9% vs. 4.5%, *p* = 0.004); all effusions were small and sheet-like, with a maximal diastolic separation of ≤10 mm, and none were associated with echocardiographic signs of cardiac tamponade. LV GLS was less negative in patients with MS than in controls (−17.49 ± 1.81% vs. −19.85 ± 1.80%, *p* < 0.001; Cohen’s d = 1.305). Patients with MS had lower aortic strain (7.54 ± 3.66% vs. 12.91 ± 3.84%, *p* < 0.001; Cohen’s d = 1.430), lower aortic distensibility (3.81 [2.23–6.06] vs. 5.95 [4.90–9.26] × 10^−3^ mmHg^−1^, *p* < 0.001) and a higher aortic stiffness index (4.77 [3.10–7.10] vs. 3.44 [2.31–4.05], *p* < 0.001). The systolic ascending aortic diameter was smaller in patients with MS (2.84 ± 0.37 vs. 3.04 ± 0.30 cm, *p* = 0.001), whereas the diastolic diameter was similar (2.64 ± 0.38 vs. 2.70 ± 0.31 cm, *p* = 0.332) (Table 2).

In a sensitivity analysis excluding all participants with pericardial effusion, the principal between-group differences remained unchanged. Among participants without pericardial effusion (53 patients with MS and 64 controls), LV GLS remained less negative in the MS group (−17.55 ± 1.81% vs. −19.90 ± 1.83%, *p* < 0.001), and aortic strain remained lower (7.65 ± 3.85% vs. 12.81 ± 3.87%, *p* < 0.001). Aortic distensibility was also lower in patients with MS (3.66 [2.38–6.06] vs. 5.94 [4.90–7.92] × 10^−3^ mmHg^−1^, *p* < 0.001), whereas the aortic stiffness index remained higher (4.80 [2.94–7.10] vs. 3.44 [2.60–4.05], *p* < 0.001). Thus, exclusion of participants with pericardial effusion did not materially alter the principal between-group findings.

Within the MS group, LV GLS was inversely correlated with aortic strain (ρ = −0.627, *p* < 0.001) and aortic distensibility (ρ = −0.506, *p* < 0.001) and positively correlated with the aortic stiffness index (ρ = 0.491, *p* < 0.001). Higher EDSS scores were associated with lower aortic strain (ρ = −0.432, *p* < 0.001) and distensibility (ρ = −0.316, *p* = 0.009), a higher stiffness index (ρ = 0.317, *p* = 0.009) and less negative LV GLS (ρ = 0.335, *p* = 0.006). Longer disease duration correlated with lower aortic strain (ρ = −0.296, *p* = 0.015), distensibility (ρ = −0.314, *p* = 0.010) and a higher stiffness index (ρ = 0.360, *p* = 0.003) but not with LV GLS (ρ = 0.182, *p* = 0.141). LDL-C correlated inversely with aortic strain (ρ = −0.356, *p* = 0.003) and distensibility (ρ = −0.487, *p* < 0.001) and positively with the stiffness index (ρ = 0.452, *p* < 0.001) and LV GLS (ρ = 0.307, *p* = 0.011). CRP was not significantly correlated with any aortic index and only weakly with LV GLS (ρ = 0.260, *p* = 0.033) (Table 3).

MS status remained independently associated with less negative LV GLS after adjustment for age, sex and BMI (B = 2.660, standardized β = 0.620, 95% CI 2.058 to 3.261, *p* < 0.001; adjusted R^2^ = 0.400) and with lower aortic strain (B = −5.724, standardized β = −0.624, 95% CI −6.931 to −4.516, *p* < 0.001; adjusted R^2^ = 0.471). MS status was associated with lower log-transformed aortic distensibility (B = −0.607, standardized β = −0.498, 95% CI −0.778 to −0.435, *p* < 0.001; adjusted R^2^ = 0.395) and a higher log-transformed aortic stiffness index (B = 0.484, standardized β = 0.454, 95% CI 0.327 to 0.641, *p* < 0.001; adjusted R^2^ = 0.336) (Table 4).

Within the MS group (Table 5), all three local aortic elastic indices remained independently associated with LV GLS in separate multivariable models adjusted for age, sex, BMI, EDSS score, and LDL-C. Higher aortic strain was associated with more negative LV GLS (B = −0.276 per 1% increase in aortic strain, standardized β = −0.558, 95% CI −0.391 to −0.161, *p* < 0.001; adjusted R^2^ = 0.378). Higher log-transformed aortic distensibility was similarly associated with more negative LV GLS (B = −1.021, standardized β = −0.356, 95% CI −1.799 to −0.242, *p* = 0.011; adjusted R^2^ = 0.229), whereas higher log-transformed aortic stiffness index was associated with less negative LV GLS (B = 1.095, standardized β = 0.339, 95% CI 0.233 to 1.956, *p* = 0.014; adjusted R^2^ = 0.224) (Table 5).

All four principal between-group echocardiographic differences remained statistically significant after Bonferroni correction (adjusted significance threshold, *p* < 0.0125). Among the exploratory within-MS correlations, most associations involving EDSS, disease duration, time since the last relapse, and LDL-C remained significant after Benjamini–Hochberg FDR adjustment. The association between CRP and LV GLS also remained significant (FDR-adjusted *p* = 0.042), whereas CRP was not associated with the aortic elastic indices after correction (Supplementary Table S2).

In sensitivity analyses with additional adjustment for cardiovascular confounders, MS status remained significantly associated with LV GLS and aortic strain after further adjustment for hyperlipidemia and systolic blood pressure (both *p* < 0.001). MS status also remained significantly associated with log-transformed aortic distensibility and aortic stiffness index after additional adjustment for hyperlipidemia (both *p* < 0.001) (Supplementary Table S1).

Intraobserver and interobserver ICCs were 0.963 and 0.980 for systolic aortic diameter and 0.954 and 0.955 for diastolic aortic diameter, respectively. Corresponding ICCs were 0.822 and 0.756 for aortic strain, 0.808 and 0.758 for aortic distensibility, and 0.829 and 0.816 for stiffness index β. ICCs with 95% confidence intervals, mean paired differences, and 95% limits of agreement are presented in Supplementary Table S3.

## 4. Discussion

In this cross-sectional case–control study, patients with MS and preserved LVEF exhibited abnormalities in local ascending aortic elasticity and LV longitudinal deformation. Compared with the control group, they had lower aortic strain and distensibility, a higher aortic stiffness index, and less negative LV GLS. MS status remained associated with all four indices after adjustment for age, sex, and BMI. Within the MS group, LV GLS correlated with each aortic index, and these associations persisted after adjustment for age, sex, BMI, EDSS score, and LDL-C. In unadjusted analyses, aortic elastic properties were also related to EDSS score, disease duration, and time since the last relapse. To our knowledge, this is the first study to characterize echocardiographically derived local ascending aortic elastic indices in MS and relate them to LV myocardial deformation within the same cohort.

Our GLS findings are consistent with previous reports of subclinical LV dysfunction in MS. Other studies have described reduced ejection fraction or abnormal tissue Doppler indices, particularly among patients with greater disability [33,34]. Using speckle-tracking, Mincu et al. found lower two- and three-dimensional LV GLS, along with reduced RV and left atrial function, in 67 patients with MS [35]. Demir et al. reported less negative GLS in relapsing–remitting MS, with a modest inverse correlation between GLS and EDSS [36]. In our cohort, mean GLS in controls (−19.85%) was close to the pooled normal mean reported in a meta-analysis of healthy subjects, whereas the mean in patients with MS (−17.49%) fell within the lower part of the normal range [37]. GLS identified a between-group difference in longitudinal function that conventional indices did not capture, consistent with its greater sensitivity [31,32]. Because reporting conventions for strain vary across studies, comparisons of association directions across the literature should be made with care [30]. Reversible reductions in LV GLS have also been described in patients with MS receiving mitoxantrone therapy, underscoring the need to account for treatment exposure in future work [53].

Evidence for vascular abnormalities in MS is more heterogeneous than that for myocardial involvement. Boshra et al. observed higher pulse wave velocity and augmentation index in relapsing–remitting MS, correlated with disease duration and disability; tonometric studies have documented higher carotid–femoral pulse wave velocity and reduced carotid compliance; and pulse contour analysis has suggested reduced large- and small-artery compliance in young patients [16,17,18,19,20]. In contrast, Mincu et al. found no significant differences in Peterson’s elastic modulus, β stiffness index, augmentation index, carotid–femoral pulse wave velocity, or flow-mediated dilation [35]. Differences in measurement techniques, vascular territories, patient characteristics, and disease burden likely explain part of this heterogeneity, as noted in the pooled analysis by Zheng et al. [15]. Our study adds local echocardiographic evidence to the literature, as aortic strain and distensibility were lower, the stiffness index was higher, and the systolic ascending aortic diameter was smaller despite a similar diastolic diameter, indicating reduced systolic expansion of the proximal aorta. Because the ascending aorta is the segment in which age-related loss of distensibility appears earliest, local indices may be sensitive to alterations in a relatively young population with low disability [50]. In this context, blood pressure did not differ between the groups, and aortic stiffening has been shown to precede, rather than follow, blood pressure elevation in the general population [54].

The central finding of this study is that, within the MS group, LV GLS was associated with all three local aortic elastic indices, and these associations persisted after adjustment for age, sex, BMI, EDSS score, and LDL-C. However, because aortic strain, distensibility, and stiffness index are mathematically related and are derived from the same underlying aortic diameter measurements, these associations should be interpreted as complementary manifestations of the same vascular phenotype rather than as independent confirmations. This observation is consistent with community-based data showing that greater aortic stiffness was independently related to worse LV GLS and with the concept that loss of aortic cushioning increases pulsatile afterload, raises myocardial oxygen demand, and reduces the diastolic transmyocardial pressure gradient, thereby affecting the subendocardial longitudinal fibers that determine GLS [14,39,40,47]. Tonometry-based studies in MS have similarly suggested altered ventricular–vascular interaction, with higher pulse pressure related to walking impairment and higher arterial and ventricular elastance despite preserved coupling [41,42]. Nevertheless, our data are cross-sectional and do not establish the direction of the relationship or a shared mechanism, as the abnormalities may reflect a common upstream process rather than a causal chain.

The mechanisms linking MS to vascular and myocardial abnormalities are likely multifactorial. Autonomic dysfunction is common in MS and may alter vascular tone and cardiac loading. Although MS is primarily a central nervous system demyelinating disease, peripheral small-fiber abnormalities have also been described in subsets of patients, including reduced intraepidermal nerve fiber density and autonomic dysfunction [55,56]. Such peripheral axonal involvement may contribute to altered neurovascular regulation and could represent an additional pathway linking MS-related immune dysregulation to vascular stiffness. However, a direct causal relationship between small-fiber loss, impaired vascular repair, and aortic stiffening has not been established and should therefore be considered hypothesis-generating. Reduced physical activity is well documented and has been shown to account for group differences in arterial function in some studies; endothelial dysfunction has been demonstrated in both the cerebral circulation and the brachial artery, where it correlates with disability; and oxidative stress and chronic systemic inflammation, integral to MS pathobiology, promote arterial stiffening through effects on the endothelium and the extracellular matrix [17,21,22,23,24,25,26,27]. In our cohort, hyperlipidemia was more prevalent in the MS group, and LDL-C was associated with all three aortic indices and with GLS in unadjusted analyses, whereas CRP was not related to the aortic indices. Adverse lipid profiles have been linked to disability progression in MS and coexist with increased arterial stiffness in MS cohorts, and they may act as mediators, confounders, or markers of a broader cardiometabolic phenotype [18,29].

Aortic elastic indices were also associated with EDSS score, disease duration, and time since the last relapse, while LV GLS was associated with EDSS score and time since the last relapse. Most of these associations remained significant after false discovery rate correction, as did the associations of LDL-C with the aortic elastic indices and LV GLS. These findings are consistent with previous reports linking GLS to disability and arterial stiffness to disease duration and neurological impairment in MS [18,36]. Nevertheless, these correlation analyses were univariable and exploratory, involved multiple correlated clinical and cardiovascular measures, and were not designed to establish independent mechanistic relationships. They should therefore be regarded as hypothesis-generating and require confirmation in larger, prospectively designed cohorts.

The coexistence of less negative LV GLS and impaired local aortic elasticity despite normal LVEF, TAPSE and sPAP indicates that a conventional echocardiographic examination may not fully characterize subclinical cardiovascular involvement in MS. Both measures can be obtained from a standard transthoracic study without additional equipment, and their combined use may provide complementary information on the myocardial and vascular components of cardiovascular risk in a research setting. Arterial stiffness predicts cardiovascular events and mortality in the general population, and MS itself carries an excess risk of macrovascular events; however, the prognostic value of the local aortic indices and of GLS in MS has not been established, and the present findings do not support routine screening [5,6,10,12]. Prospective studies are required to determine whether these measures identify patients who would benefit from intensified management of modifiable risk factors.

The higher prevalence of small pericardial effusion in the MS group may reflect a broader immune-mediated or inflammatory milieu. Autoimmune comorbidities are relatively common in people with MS, with increased frequencies of several immune-mediated disorders, including autoimmune thyroid disease, inflammatory bowel disease, and psoriasis, reported in systematic reviews and meta-analyses [57,58]. However, a direct association between MS itself and pericardial effusion has not been established, and the etiology of these small effusions therefore remains uncertain in the present cohort. Importantly, exclusion of participants with pericardial effusion did not materially alter the differences in LV GLS or local aortic elastic indices.

Several limitations should be noted. First, the single-center, cross-sectional design precludes inferences regarding temporality or causality. Second, the sample size was modest, and the multivariable models within the MS group included several covariates relative to the number of participants; therefore, these estimates should be interpreted cautiously. In addition, the cohort consisted exclusively of patients with relapsing–remitting MS and predominantly mild disability, limiting generalizability to progressive MS phenotypes and more advanced disease. Third, although the groups were age-matched, they were recruited from different clinical settings, were not sex-matched, and differed in BMI and the prevalence of hyperlipidemia. Additional sensitivity analyses accounting for hyperlipidemia and systolic blood pressure did not materially alter the principal findings; nevertheless, residual confounding by unmeasured cardiovascular and lifestyle factors, including physical activity, cannot be excluded. Fourth, the aortic elastic indices were derived from local M-mode measurements combined with brachial rather than central blood pressure; they depend on measurement level, edge convention, and cardiac-cycle timing and are not interchangeable with carotid–femoral pulse wave velocity or magnetic resonance-derived measures of aortic stiffness [13]. Agreement was higher for aortic diameters than for the derived elasticity indices. The Bland–Altman limits of agreement indicate variability between readings that should be considered when interpreting individual measurements. Fifth, the original image acquisition and analysis were performed with knowledge of group allocation, which may have introduced observer bias, despite the subsequent reproducibility assessment. Finally, the potential effects of disease-modifying therapies, previous corticosteroid exposure, and other unmeasured cardiovascular or immune-mediated factors could not be fully addressed, and the underlying mechanistic pathways were not directly investigated.

## 5. Conclusions

Patients with MS and preserved LVEF exhibited lower ascending aortic strain and distensibility, a higher aortic stiffness index, and less negative LV GLS than age-matched controls. MS status remained associated with these indices after adjustment for age, sex, and BMI. Within the MS group, LV GLS was independently associated with all three local aortic elastic indices, regardless of demographic, anthropometric, disability-related, and lipid covariates. These findings indicate that subclinical myocardial and proximal aortic abnormalities coexist and are interrelated in MS, even at a stage of minimal disability. Longitudinal studies incorporating complementary measures of arterial stiffness, treatment exposure, and clinical outcomes are required to clarify the temporal sequence, mechanisms, and prognostic significance of these observations.

## Figures and Tables

**Table 1:** Baseline demographic, clinical, MS-related and laboratory characteristics of the study population.

Variable	Controls (*n* = 67)	MS (*n* = 67)	*p* Value
**Demographic and clinical characteristics**			
Age, years	42.36 ± 9.67	41.51 ± 10.80	0.632 ^a^
Female sex, *n* (%)	44 (65.7)	58 (86.6)	**0.005** ^c^
Body mass index, kg/m^2^	24.9 (23.3–28.7)	24.1 (22.3–26.6)	**0.020** ^b^
Systolic blood pressure, mmHg	115 (109–125)	113 (107–123)	0.251 ^b^
Diastolic blood pressure, mmHg	77.90 ± 8.52	78.84 ± 9.42	0.546 ^a^
Hypertension, *n* (%)	11 (16.4)	12 (17.9)	0.819 ^c^
Diabetes mellitus, *n* (%)	1 (1.5)	3 (4.5)	0.619 ^d^
Current smoking, *n* (%)	22 (32.8)	25 (37.3)	0.587 ^c^
Hyperlipidemia, *n* (%)	5 (7.5)	16 (23.9)	**0.009** ^c^
Antihypertensive medication, *n* (%)	11 (16.4)	10 (14.9)	1.000 ^d^
Statin use, *n* (%)	5 (7.5)	4 (6.0)	1.000 ^d^
**MS-related characteristics**			
Disease phenotype (RRMS/SPMS/PPMS), *n* (%)	—	RRMS, 67 (100)	—
Disease duration, years	—	12.0 (5.0–20.0)	—
Time since last relapse, years	—	3.0 (1.0–9.0)	—
EDSS score	—	1.0 (1.0–2.0)	—
Previous corticosteroid treatment for relapse, *n* (%)	—	61 (91.0)	—
**Current disease-modifying therapy, *n* (%)**			
Pyrimidine synthesis inhibitor	—	15 (22.4)	—
Fumarate	—	12 (17.9)	—
S1P receptor modulator	—	12 (17.9)	—
Anti-CD20 monoclonal antibody	—	9 (13.4)	—
Purine analogue	—	8 (11.9)	—
Interferon-based therapy	—	6 (9.0)	—
Anti-α4-integrin monoclonal antibody	—	2 (3.0)	—
Glatiramer acetate	—	1 (1.5)	—
No current DMT	—	2 (3.0)	—
**Laboratory parameters (MS group)**			
LDL-C, mg/dL	—	122.2 ± 40.6	—
HDL-C, mg/dL	—	62 (49–70)	—
Triglycerides, mg/dL	—	88 (72–123)	—
Glucose, mg/dL	—	85 (81–92)	—
HbA1c, %	—	5.30 (5.20–5.60)	—
Creatinine, mg/dL	—	0.70 (0.60–0.80)	—
Hemoglobin, g/dL	—	13.78 ± 1.39	—
C-reactive protein, mg/L	—	1.70 (0.70–3.30)	—
TSH, mIU/L	—	1.70 (1.10–2.70)	—

Data are presented as mean ± standard deviation, median (interquartile range) or *n* (%). ^a^ Independent-samples Student’s *t*-test; ^b^ Mann–Whitney U test; ^c^ Pearson’s chi-square test; ^d^ Fisher’s exact test. Bold values indicate *p* < 0.05. EDSS: Expanded Disability Status Scale; HbA1c: glycated hemoglobin; HDL-C: high-density lipoprotein cholesterol; LDL-C: low-density lipoprotein cholesterol; MS: multiple sclerosis; PPMS: primary progressive MS; RRMS: relapsing–remitting MS; SPMS: secondary progressive MS; TSH: thyroid-stimulating hormone.

**Table 2:** Echocardiographic parameters, left ventricular global longitudinal strain and local ascending aortic elastic properties.

Parameter	Controls (*n* = 67)	MS (*n* = 67)	*p* Value
**Left ventricular structure and function**			
LVEF, %	62.0 (59.0–67.0)	65.0 (59.0–67.0)	0.872 ^b^
LV GLS, %	−19.85 ± 1.80	−17.49 ± 1.81	**<0.001** ^a^
LVEDD, cm	4.30 (4.10–4.70)	4.10 (4.00–4.30)	**<0.001** ^b^
LVESD, cm	2.30 (2.10–2.70)	2.10 (2.00–2.30)	**<0.001** ^b^
LAVI, mL/m^2^	14.20 (12.00–17.70)	13.67 (11.20–16.30)	0.507 ^b^
LVMI, g/m^2^	71.50 (63.50–90.40)	69.10 (61.60–77.80)	0.160 ^b^
**Right ventricular function and pulmonary pressure**			
TAPSE, cm	1.99 ± 0.19	1.98 ± 0.24	0.776 ^a^
RV S′, cm/s	11.99 ± 1.55	12.02 ± 1.52	0.906 ^a^
sPAP, mmHg	18.91 ± 4.89	18.55 ± 5.17	0.681 ^a^
**Ascending aorta and local elastic indices**			
Systolic aortic diameter (AoS), cm	3.04 ± 0.30	2.84 ± 0.37	**0.001** ^a^
Diastolic aortic diameter (AoD), cm	2.70 ± 0.31	2.64 ± 0.38	0.332 ^a^
Aortic strain, %	12.91 ± 3.84	7.54 ± 3.66	**<0.001** ^a^
Aortic distensibility, ×10^−3^ mmHg^−1^	5.95 (4.90–9.26)	3.81 (2.23–6.06)	**<0.001** ^b^
Aortic stiffness index (β)	3.44 (2.31–4.05)	4.77 (3.10–7.10)	**<0.001** ^b^
**Other findings**			
Pericardial effusion, *n* (%)	3 (4.5)	14 (20.9)	**0.004** ^c^

Data are presented as mean ± standard deviation, median (interquartile range) or *n* (%). ^a^ Independent-samples Student’s *t*-test; ^b^ Mann–Whitney U test; ^c^ Pearson’s chi-square test. Bold values indicate *p* < 0.05. Between-group effect sizes (Cohen’s d) were 1.305 for LV GLS and 1.430 for aortic strain. GLS: global longitudinal strain; LAVI: left atrial volume index; LV: left ventricular; LVEDD: LV end-diastolic diameter; LVEF: LV ejection fraction; LVESD: LV end-systolic diameter; LVMI: LV mass index; MS: multiple sclerosis; RV S′: right ventricular tissue Doppler systolic velocity; sPAP: systolic pulmonary artery pressure; TAPSE: tricuspid annular plane systolic excursion.

**Table 3:** Spearman correlations of local ascending aortic elastic indices and LV GLS.

Variable	Aortic Strain, ρ (*p*)	Aortic Distensibility, ρ (*p*)	Aortic Stiffness Index (β), ρ (*p*)	LV GLS, ρ (*p*)
LV GLS	−0.627 (**<0.001**)	−0.506 (**<0.001**)	0.491 (**<0.001**)	—
EDSS score	−0.432 (**<0.001**)	−0.316 (**0.009**)	0.317 (**0.009**)	0.335 (**0.006**)
Disease duration	−0.296 (**0.015**)	−0.314 (**0.010**)	0.360 (**0.003**)	0.182 (0.141)
Time since last relapse	−0.354 (**0.003**)	−0.312 (**0.010**)	0.329 (**0.006**)	0.338 (**0.005**)
LDL-C	−0.356 (**0.003**)	−0.487 (**<0.001**)	0.452 (**<0.001**)	0.307 (**0.011**)
C-reactive protein	−0.221 (0.072)	−0.108 (0.386)	0.080 (0.520)	0.260 (**0.033**)

Values are Spearman’s rank correlation coefficients (ρ) with two-sided *p* values; bold values indicate *p* < 0.05. EDSS: Expanded Disability Status Scale; GLS: global longitudinal strain; LDL-C: low-density lipoprotein cholesterol; LV: left ventricular; MS: multiple sclerosis.

**Table 4:** Multivariable linear regression models of the association of MS status with LV GLS and local ascending aortic elastic indices.

Dependent Variable	B	Standardized β	95% CI for B	*p* Value	Adjusted R^2^
LV GLS, %	2.660	0.620	2.058 to 3.261	<0.001	0.400
Aortic strain, %	−5.724	−0.624	−6.931 to −4.516	<0.001	0.471
ln(aortic distensibility)	−0.607	−0.498	−0.778 to −0.435	<0.001	0.395
ln(aortic stiffness index β)	0.484	0.454	0.327 to 0.641	<0.001	0.336

Each row is a separate model with MS status as the principal independent variable, adjusted for age, sex and body mass index. Aortic distensibility and aortic stiffness index were natural-log-transformed due to skewed distributions; their coefficients are interpreted on the logarithmic scale. CI: confidence interval; GLS: global longitudinal strain; LV: left ventricular; MS: multiple sclerosis.

**Table 5:** Multivariable linear regression models of the associations between LV GLS and local ascending aortic elastic indices within the MS group.

Model	Dependent Variable	Independent Variable of Interest	B	Standardized β	95% CI for B	*p* Value	Adjusted R^2^
1	LV GLS, %	Aortic strain, per 1%	−0.276	−0.558	−0.391 to −0.161	<0.001	0.378
2	LV GLS, %	ln(aortic distensibility)	−1.021	−0.356	−1.799 to −0.242	0.011	0.229
3	LV GLS, %	ln(aortic stiffness index β)	1.095	0.339	0.233 to 1.956	0.014	0.224

All models are adjusted for age, sex, body mass index, EDSS score, and LDL-C. B, unstandardized coefficient; β, standardized coefficient. LV GLS was analyzed using the signed convention, with myocardial shortening represented by negative values. Accordingly, in Model 1, a negative coefficient indicates more negative (better) GLS with increasing aortic strain; in Model 2, higher log-transformed aortic distensibility is associated with more negative (better) GLS; and in Model 3, higher log-transformed aortic stiffness index is associated with less negative (worse) GLS. Aortic distensibility and stiffness index were natural-log-transformed because of their skewed distributions. CI, confidence interval; EDSS, Expanded Disability Status Scale; GLS, global longitudinal strain; LDL-C, low-density lipoprotein cholesterol; LV, left ventricular; MS, multiple sclerosis.

## Data Availability

The data presented in this study are available on reasonable request from the corresponding author. The data are not publicly available because they contain information that could compromise the privacy of research participants.

## Supplementary Materials

The following supporting information can be downloaded at: https://www.mdpi.com/article/10.3390/diagnostics16203385/s1, Table S1: Sensitivity analyses of the association between MS status and LV GLS and local ascending aortic elastic indices after additional adjustment for cardiovascular confounders; Table S2: Benjamini–Hochberg false discovery rate adjustment of exploratory correlations between clinical/laboratory variables and LV GLS and local ascending aortic elastic indices within the MS group; Table S3: Intraobserver and interobserver reproducibility of aortic diameters and derived aortic elasticity indices in 30 participants.

## Abbreviations

The following abbreviations are used in this manuscript:

2D-STE	two-dimensional speckle-tracking echocardiography
AoD	diastolic ascending aortic diameter
AoS	systolic ascending aortic diameter
BMI	body mass index
CI	confidence interval
CRP	C-reactive protein
DBP	diastolic blood pressure
EDSS	Expanded Disability Status Scale
GLS	global longitudinal strain
IQR	interquartile range
LAVI	left atrial volume index
LDL-C	low-density lipoprotein cholesterol
LV	left ventricular
LVEF	left ventricular ejection fraction
LVMI	left ventricular mass index
MS	multiple sclerosis
RV	right ventricular
SBP	systolic blood pressure
SD	standard deviation
sPAP	systolic pulmonary artery pressure
TAPSE	tricuspid annular plane systolic excursion
