# Supplementary material for: Association Between Local Ascending Aortic Elastic Properties and Left Ventricular Global Longitudinal Strain in Patients with Multiple Sclerosis: A Cross-Sectional Case–Control Echocardiographic Study
Source: Diagnostics (Basel). 2026 Oct 18;16(20):3385. doi: 10.3390/diagnostics16203385 (PMC13702874; doi:10.3390/diagnostics16203385)
Supplement: Supplementary file 1 [file diagnostics-16-03385-s001.zip › diagnostics-4545643-supplementary.pdf]

Supplementary Materials

Supplementary Table S1. Sensitivity analyses of the association between MS status and LV GLS and local ascending aortic elastic indices after additional adjustment for cardiovascular confounders.

Dependent variable	Additional covariates	B for MS status	Standardized β	95% CI	*p*	Adjusted R^2
LV GLS, %	Age, sex, BMI, hyperlipidemia, SBP	2.593	0.604	1.958 to 3.229	<0.001	0.394
Aortic strain, %	Age, sex, BMI, hyperlipidemia, SBP	−5.592	−0.609	−6.864 to −4.320	<0.001	0.469
ln(aorticdistensibility)	Age, sex, BMI, hyperlipidemia	−0.554	−0.455	−0.732 to −0.376	<0.001	0.407
ln(aortic stiffnessindex β)	Age, sex, BMI, hyperlipidemia	0.430	0.404	0.268 to 0.592	<0.001	0.355

Note: Each row represents a separate multivariable linear regression model with MS status as the principal independent variable. Models for LV GLS and aortic strain were adjusted for age, sex, body mass index, hyperlipidemia, and systolic blood pressure. Models for aortic distensibility and aortic stiffness index were adjusted for age, sex, body mass index, and hyperlipidemia. Systolic blood pressure was not additionally entered into the latter two models because systolic and diastolic blood pressure are mathematical components of these indices. Aortic distensibility and stiffness index were natural-log-transformed because of their skewed distributions. B, unstandardized coefficient; β, standardized coefficient; BMI, body mass index; CI, confidence interval; GLS, global longitudinal strain; LV, left ventricular; MS, multiple sclerosis; SBP, systolic blood pressure.

Supplementary Table S2. Benjamini–Hochberg false discovery rate adjustment of exploratory correlations between clinical/laboratory variables and LV GLS and local aortic elastic indices within the MS group.

**Variable**	**Outcome**	**Spearman ϱ**	**Raw p value**	**FDR-adjusted p value**
EDSS score	Aortic strain	−0.432	<0.001	0.0018
EDSS score	Aortic distensibility	−0.316	0.009	0.0156
EDSS score	Aortic stiffness index (β)	0.317	0.009	0.0156
EDSS score	LV GLS	0.335	0.006	0.0139
Disease duration	Aortic strain	−0.296	0.015	0.0201
Disease duration	Aortic distensibility	−0.314	0.010	0.0156
Disease duration	Aortic stiffness index (β)	0.360	0.003	0.0110
Disease duration	LV GLS	0.182	0.141	0.1562
Time since last relapse	Aortic strain	−0.354	0.003	0.0110
Time since last relapse	Aortic distensibility	−0.312	0.010	0.0156
Time since last relapse	Aortic stiffness index (β)	0.329	0.006	0.0144
Time since last relapse	LV GLS	0.338	0.005	0.0139
LDL-C	Aortic strain	−0.356	0.003	0.0110
LDL-C	Aortic distensibility	−0.487	<0.001	0.0006
LDL-C	Aortic stiffness index (β)	0.452	<0.001	0.0012
LDL-C	LV GLS	0.307	0.011	0.0162
C-reactive protein	Aortic strain	−0.221	0.072	0.0849
C-reactive protein	Aortic distensibility	−0.108	0.386	0.4058
C-reactive protein	Aortic stiffness index (β)	0.080	0.520	0.5198
C-reactive protein	LV GLS	0.260	0.033	0.0418

Note: Values are Spearman's rank correlation coefficients (ϱ) with two-sided raw and Benjamini–Hochberg false discovery rate (FDR)-adjusted p values. The FDR correction was applied across the 20 exploratory correlations shown in the table. FDR-adjusted p values < 0.05 are shown in bold. LV GLS was analyzed using the signed convention, with myocardial shortening represented by negative values. EDSS, Expanded Disability Status Scale; FDR, false discovery rate; GLS, global longitudinal strain; LDL-C, low-density lipoprotein cholesterol; LV, left ventricular; MS, multiple sclerosis.

Supplementary Table S3. Intraobserver and interobserver reproducibility of aortic diameters and derived aortic elasticity indices in 30 participants.

**Parameter**	**Comparison**	**ICC (95% CI)**	**Mean paired difference**	**95% limits of agreement**
Systolic aortic diameter, cm	Intraobserver	0.963 (0.917–0.980)	−0.060	−0.182 to 0.062
	Interobserver	0.980 (0.963–0.988)	−0.013	−0.137 to 0.110
Diastolic aortic diameter, cm	Intraobserver	0.954 (0.879–0.977)	−0.040	−0.182 to 0.102
	Interobserver	0.955 (0.905–0.971)	−0.043	−0.186 to 0.099
Aortic strain, %	Intraobserver	0.822 (0.631–0.913)	−0.621	−5.868 to 4.627
	Interobserver	0.756 (0.556–0.885)	1.204	−4.859 to 7.267
Aortic distensibility, $\times 10^{-3}$ mmHg^{-1}	Intraobserver	0.808 (0.603–0.914)	−0.281	−2.196 to 1.635
	Interobserver	0.758 (0.534–0.882)	0.373	−1.858 to 2.604
Aortic stiffness index β	Intraobserver	0.829 (0.490–0.967)	0.850	−4.064 to 5.765
	Interobserver	0.816 (0.506–0.951)	−0.376	−5.303 to 4.550

Note: ICC, intraclass correlation coefficient; CI, confidence interval. All comparisons included 30 participants. ICCs represent single-measure, two-way absolute-agreement estimates [ICC(A,1)]. The 95% CIs were calculated using 10,000 participant-level bootstrap resamples. Intraobserver comparisons represent the same observer’s repeat reading versus the initial reading; interobserver comparisons represent the second observer’s reading versus the first observer’s initial reading. Paired differences were calculated as repeat or second-observer reading minus initial reading. The 95% limits of agreement were calculated as the mean paired difference ± 1.96 SD. Differences in aortic strain are expressed in percentage points.
